# Highlights of Medial Tibial Stress Syndrome in Military Recruits: A Narrative Review

**DOI:** 10.7759/cureus.75376

**Published:** 2024-12-09

**Authors:** Mohammed Alessa, Yazeed O Almutairi, Mohammed Alquhayz, Abdullah Alothman, Fahad Alajlan, Alhanoof Alajlan, Nasser M AbuDujain, Hamza M Alrabai

**Affiliations:** 1 Department of Family and Community Medicine, Security Forces Hospital, Riyadh, SAU; 2 Department of Family and Community Medicine, King Saud University Medical City, Riyadh, SAU; 3 Department of Medical Services, Royal Saudi Naval Forces, Ministry of Defense, Riyadh, SAU; 4 Department of Family and Community Medicine, King Fahad Medical City, Riyadh, SAU; 5 Department of Anesthesiology, King Saud University Medical City, Riyadh, SAU; 6 College of Medicine, King Saud University Medical City, Riyadh, SAU; 7 Department of Orthopedics, King Saud University, Riyadh, SAU

**Keywords:** armed forces, medial tibial stress syndrome, military, navy, shin splint

## Abstract

Medial tibial stress syndrome (MTSS), commonly known as shin splints, is characterized by pain and inflammation in the shin caused by repetitive stress. While often associated with sports and physical activity, MTSS can significantly impact daily life, particularly in military recruits, making it a pertinent concern for this population. This narrative review synthesizes findings from a comprehensive search of databases to explore the prevalence, risk factors, and management of MTSS among military recruits. The search identified 35 studies discussing these aspects. MTSS is highly prevalent among military recruits, with an incidence ranging from 35% to 56%. Key risk factors include female sex, higher BMI, low aerobic fitness, smoking, and specific anatomical characteristics. Various treatments, including extracorporeal shockwave therapy (ESWT), compression therapy, pneumatic leg braces, and shock-absorbing insoles, have been shown to effectively accelerate recovery. Early identification of at-risk individuals could significantly reduce MTSS incidence and related healthcare costs. Emerging artificial intelligence tools also hold promise for delivering precise risk assessments. In conclusion, MTSS is a common issue among military personnel, driven by the physical demands of their training and specific risk factors. Further research into predictors of MTSS across diverse military populations is essential to improve outcomes.

## Introduction and background

Medial tibial stress syndrome (MTSS), or shin splints, is inflammation and pain in the middle and lower part of the tibia (specifically, along the posteromedial border). This condition is typically associated with physical activity, exercise, and sports but excludes pain resulting from ischemia or stress fractures [1,2]. The pathophysiology of MTSS is characterized by repetitive mechanical stress on the tibia, leading to microtrauma in the muscles, tendons, and periosteum, particularly in the posterior tibialis and flexor digitorum longus [1,3,4]. This stress induces inflammatory responses, releasing cytokines that cause pain, swelling, and tenderness along the shin. Over time, continued overload without proper recovery can result in periostitis, stress fractures, and chronic pain, as the tissues fail to adequately repair and adapt [1-3]. This combination of biomechanical stress and inflammatory damage contributes to the development and persistence of MTSS.

MTSS usually carries a favorable prognosis but can become chronic and debilitating [3]. High-impact exercises and increased loading significantly increase the risk of MTSS and other bone stress injuries. Intrinsic risk factors for MTSS include female sex, a history of MTSS, high body mass index (BMI), and specific biomechanical characteristics such as flat feet (pes planus), limited ankle dorsiflexion flexibility (typically less than 20 degrees), and altered hip rotation range [4]. For example, excessive internal hip rotation or limited external rotation has been associated with abnormal loading patterns on the tibia, contributing to the development of MTSS [4,5]. These biomechanical traits may exacerbate stress on the medial tibial region during repetitive activities like running or marching [4,5]. Among military personnel, extrinsic factors contributing to MTSS include the demands of long military marches and physical activities involving excessive leg training [6]. These situational stressors, combined with intrinsic risk factors such as biomechanics or body composition, significantly increase the risk of developing MTSS in this population [6].

MTSS is a common source of leg pain among military personnel, and symptoms can develop as soon as two to three months into training [7,8]. A broad spectrum of treatment options for MTSS exists, ranging from mere rest to surgical intervention [9]. Given the high prevalence of MTSS among military personnel, it significantly impacts operational readiness, causing delays in training and deployment while also driving up healthcare costs due to extended recovery times, repeated medical interventions, and long-term treatment needs.

Therefore, this review assesses the characteristics, predictors, and risk factors of developing MTSS among military recruits. While MTSS is well-documented in sports medicine, there remains a lack of focused research on military recruits, a unique population with specific risk factors related to rigorous training, physical demands, and environmental stressors. This review aims to synthesize existing evidence and provide insights into how intrinsic and extrinsic factors specifically interact in military settings, offering a more tailored understanding of MTSS in this context and identifying areas for improved prevention and management strategies.

## Review

Methods

This is a narrative review aimed at synthesizing existing literature on MTSS among military personnel. We qualitatively analyze and summarize relevant studies to identify trends, risk factors, and management strategies.

Search Strategy and Sources

In January 2024, we conducted a comprehensive search of several databases, including the Cochrane Database of Systematic Reviews, Google Scholar, ERIC, Embase, and PubMed Medline. We used the following search terms: "medial tibial stress syndrome", "military personnel", "shin splints", "tibial stress fractures", risk factors", "prevalence", and "treatment", combined with relevant Medical Subject Headings (MeSH). In addition, we reviewed the reference lists of relevant articles and reviews to identify further sources. A follow-up search conducted in September 2024 revealed no additional papers of interest.

Inclusion and Exclusion Criteria

We included studies that specifically addressed MTSS among military personnel, including interventional studies, cohort studies (both prospective and retrospective), review articles, and published theses. Studies with no time restrictions were considered as long as they provided relevant data. We also included studies on tibial stress fractures within military populations.

We excluded studies focusing on athletes or the general population, letters to the editor, non-English articles, and those with restricted access.

Data Extraction

The data were extracted from the included studies using a standardized extraction form. For each study, we recorded key details such as the primary author's last name, study type, publication year, geographical location (city or country), and significant findings. Since this is a narrative review, we synthesized the findings qualitatively to identify common themes, trends, and gaps in the existing literature regarding MTSS among military personnel.

Results

**Figure 1:**
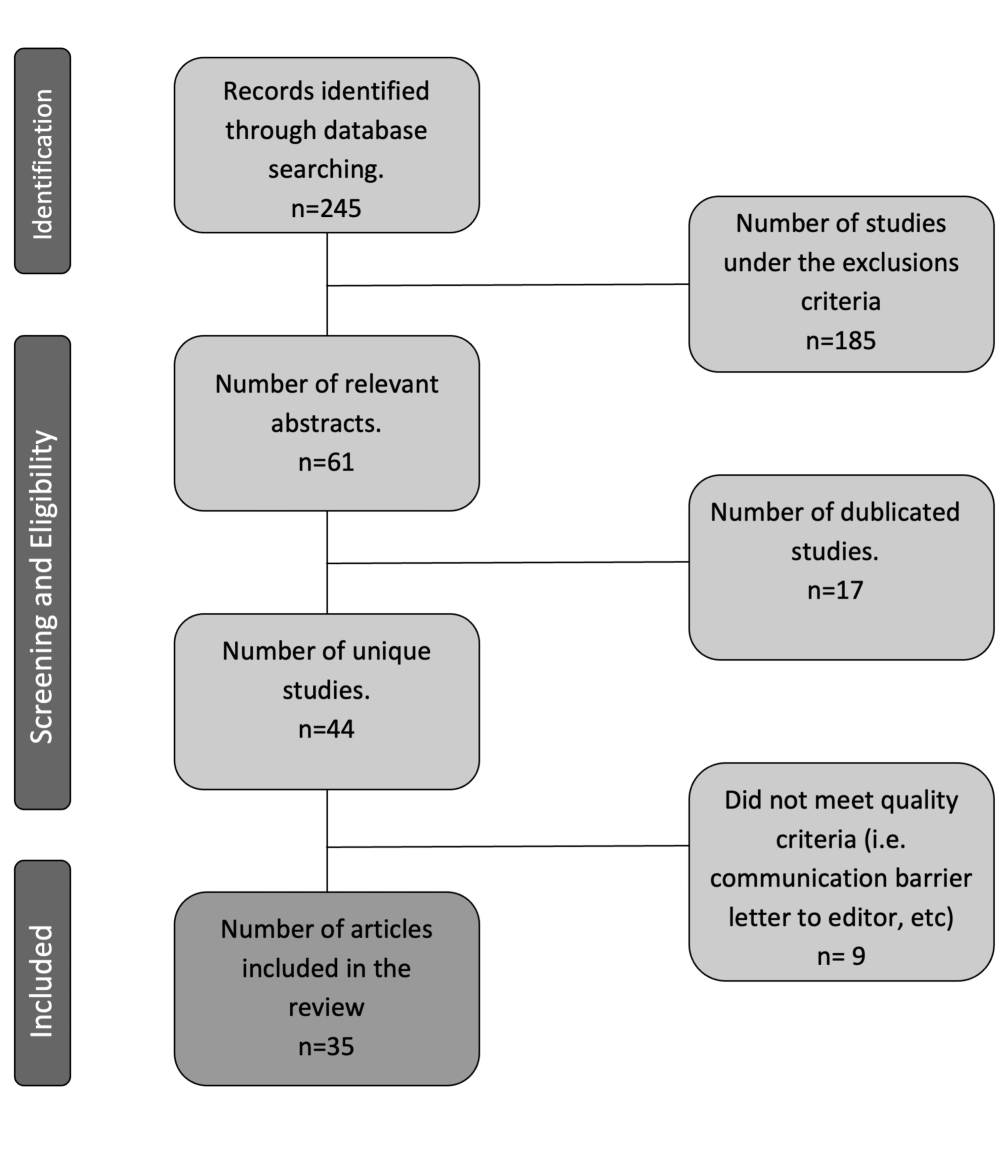
Flow diagram of literature screening and selection process

Our search yielded 245 studies. In phase 1, abstract screening was carried out to identify studies relevant to the topic of interest; after excluding the irrelevant and duplicated papers, the number was reduced to 44. All articles were compiled into a single spreadsheet. Papers involving athletes and non-military individuals were excluded, reaching a final number of 35 papers discussed in the review (Figure 1).

Prevalence, Incidence, Clinical Aspects, and Severity

The incidence rate of MTSS is 50-56% [10,11], and tibial periostitis, a subtype of MTSS, develops in 4-10% of recruits after 8-12 weeks of basic training [12]. MTSS significantly impacts training time, contributing to 19.8% of all rehabilitation days [13]. The financial burden of training-related injuries is substantial, with the US government projected to spend $31,000 per recruit annually on operations and medical care [11].

As reported by Jakayla Campbell, the prevalence of MTSS among army officers included around 56% of the included sample during their training program, with more than 50% having a history of MTSS [11]. Moreover, there was an incidence of 5.67% after a 26-week combat infantryman's course [13]. In terms of clinical aspects and causes, Milgrom et al. found that nearly half of the recruits, regardless of their pre-army sport participation, developed exertional pain along the medial tibial border just after 14 weeks of basic training [14].

Risk Factors

As demonstrated in Table 1, female sex is a risk factor in developing MTSS [2,15], which may be because of the associated smaller tibial cross-sectional dimensions, greater hip range of motion, and distinct lower extremity mechanics during running. Other risk factors are obesity, ankle plantar flexion range of motion, and hip external rotation range of motion [2,16]. Bonanno et al. found that identifying pronated feet before training reduces the incidence of MTSS and enables earlier intervention [2]. Low aerobic fitness, smoking, and increased medial foot pressure have been identified as risk factors for the development of MTSS among military personnel [17-19]. Compared to the general non-military population, recruits undergo extensive travel by foot in general during their basic training, especially in the initial weeks when they are not fully adapted. This considerable distance covered is believed to be a significant risk factor contributing to the prevalent occurrence of overuse injuries witnessed during their training [17].

**Table 1:** Key risk factors for medial tibial stress syndrome MTSS: medial tibial stress syndrome; UK: United Kingdom

Risk factor	Reference/literature	Study design	Results
Female sex	Yates and White, 2004, Australia	Randomized clinical trial	Females have a higher risk of MTSS due to smaller tibial cross-sectional dimensions, greater hip range of motion, and distinct lower-extremity mechanics during running.
Obesity	Hamstra-Wright et al., 2015	Systematic review	Individuals with MTSS had significantly higher BMIs than the control group. (mean difference (MD)=0.79, 95% CI 0.38 to 1.20).
Ankle plantarflexion range of motion	Hamstra-Wright et al., 2015	Systematic review	Individuals with MTSS showed a 5.94° increase in plantar flexion compared to the control group.
Hip external rotation range of motion	Hamstra-Wright et al., 2015	Systematic review	A higher occurrence of MTSS was linked to increased hip external rotation.
Navicular drop	Yates and White, 2004, Australia	Randomized clinical trial	Individuals with MTSS exhibited a navicular drop that was 1.19 mm more pronounced than those without MTSS.
Low aerobic fitness	Sharma et al., 2010, UK	Prospective study	Recruits exhibiting lower fitness (as measured by aerobic capacity tests) levels were 3.6 times more likely to develop MTSS.
Smoking	Sharma et al., 2010, UK	Prospective study	When combined with other factors, such as low aerobic fitness and medial foot pressure, smoking was also identified as a contributing factor to increased injury risk.
Medial foot pressure	Sharma et al., 2010, UK	Prospective study	An imbalance with greater medial foot pressure was a primary risk factor for developing MTSS.
Long distance travel	Whittle, 2022, UK	Prospective cohort	A correlation existed between long distances traveled by foot and the symptomatic manifestation of lower-limb overuse injury two weeks later.

Investigations and Diagnosis

MTSS can be diagnosed effectively through a patient's medical history and physical examination, with a high degree of agreement among clinicians [20]. Since imaging tests like X-rays and bone scans do not effectively distinguish between athletes with and without MTSS, they are not recommended for confirming this diagnosis unless there is suspicion of other conditions such as tibial stress fractures or osteosarcoma, where imaging would be a logical step [20,21]. Also, MRI may be useful in certain cases to assess soft tissue damage or to rule out other potential causes of pain, but it is not routinely required for the diagnosis of MTSS [20,21].

Treatment

Treatment for MTSS among military recruits encompasses various methods, including extracorporeal shockwave therapy (ESWT), compression therapy, the fascial distortion model, pneumatic leg braces, and shock-absorbing insoles [22]. ESWT, in particular, outshines the others in effectiveness [23], and its effectiveness is further enhanced when paired with specific exercise regimens, significantly speeding up clinical and functional recovery [24]. As highlighted in a study by Shamsi Majelan and Fadaei Dehcheshmeh [25], ESWT improves function and alleviates discomfort in MTSS patients. Combining ESWT with conventional treatment yields better outcomes (running program completion time and general satisfaction with management) than standard treatment alone [26]. Compression therapy provides moderate benefits and aids military personnel in resuming training [27]. The fascial distortion model provides quick pain relief and restoration of exercise tolerance despite some patients experiencing high pain intensity during treatment [28]. In contrast, pneumatic leg braces show negligible additional benefits in general satisfaction, physical endurance, and Sports Activity Rating Scale scores [29]. Shock-absorbing insoles, supported by level I evidence for preventing MTSS [30], potentially gain further effectiveness when used alongside exercises, manual methods, or compression stockings [31,32].

Prevention and Prediction

The occurrence of MTSS in the United States among Army Reserve Officer Training Corps cadets demonstrates a correlation between MTSS and military practices [11]. Recent research underscores the significance of investigating diverse interventions to address MTSS incidence among this group, including gait retraining, agility training, and resistance training [33,34]. Notably, a successful reduction in MTSS incidence was observed in an at-risk military sample by implementing a gait retraining program [35]. Conversely, an exercise program emphasizing muscular strengthening, coordination, and flexibility training did not demonstrably reduce the risk of MTSS in individuals engaging in increased physical activity. Additionally, prefabricated foot orthoses significantly decrease lower-limb overuse injuries, including MTSS, which suggests that foot orthoses may prevent injuries during military training [36].

In exploring machine-learning approaches for predicting MTSS risk, methods such as naive Bayes, ensemble, and support vector machines stand out for their effectiveness [37]. A particularly robust predictive model emerges when integrating variables like sex, past MTSS experiences, and hip external rotation, offering a potent tool for pinpointing individuals at high risk [38]. Additionally, when combined, shin palpation, shin edema tests, and female sex reliably predict MTSS symptoms in physically active, asymptomatic individuals [39]. Notably, the calibrated random-forest model accurately forecasts MTSS onset [40]. These insights highlight the effectiveness of machine-learning methods and pave the way for more precise and individualized MTSS risk assessments.

## Conclusions

MTSS is common among those who exert prolonged or high-intensity physical activity. MTSS is associated with high BMI, female sex, and some anatomical variations, like navicular drop (pronated feet). Highlighting the impact of this condition on military recruits is vital for better and earlier detection and treatment. Future research investigating predictors of MTSS among military personnel from different fields and ethnicities is crucial for better outcomes.
